# The Impact of Teamwork Climate on Hospital Employees’ Job Satisfaction and Perceived Healthcare Quality

**DOI:** 10.3390/healthcare14101259

**Published:** 2026-05-07

**Authors:** Mao-Hung Liao, Yafang Tsai, Shih-Wang Wu, Chien-Ying Lee, Shwn-Huey Shieh

**Affiliations:** 1 Office of Vice Superintendent, Yonghe Cardinal Tien Hospital, New Taipei City 234, Taiwan; mhl5488@gmail.com; 2 Department of Health Policy and Management, Chung Shan Medical University, Taichung 402, Taiwan; 3 Department of Medical Management, Chung Shan Medical University Hospital, Taichung 402, Taiwan; 4 Department of Hospital and Health Care Administration, Chia Nan University of Pharmacy & Science, Tainan 717, Taiwan; 5 Department of Medical, Chung Shan Medical University, Taichung 402, Taiwan; cshd015@csmu.edu.tw; 6 Department of Health Services Administration, China Medical University, Taichung 406, Taiwan; shshieh@mail.cmu.edu.tw

**Keywords:** healthcare professional, team collaboration, teamwork, job satisfaction, quality of care

## Abstract

**Background:** Interdisciplinary teamwork is widely recognized as a critical component of high-quality healthcare delivery. Healthcare services are delivered through interactions between healthcare professionals and patients; employees’ attitudes and behaviors may play a pivotal role in determining healthcare quality. **Purpose:** This study aimed to examine the effects of teamwork climate on employees’ job satisfaction and perceived healthcare quality. **Methods:** A cross-sectional survey design was employed to assess hospital employees’ perceptions of teamwork climate, job satisfaction, and healthcare quality. We adopted a structured questionnaire to collect data. A total of 528 questionnaires were distributed, and 462 valid responses were obtained, yielding a valid response rate of 87.5%. Data were analyzed using SPSS version 22.0. Descriptive statistics and one-way analysis of variance (ANOVA) were conducted to examine differences across demographic characteristics. Correlation analyses were performed to evaluate the relationships among teamwork climate, job satisfaction, and healthcare quality. **Results:** The findings indicated that teamwork climate was positively associated with perceived healthcare quality (r = 0.4333, *p* < 0.001) and job satisfaction (r = 0.695, *p* < 0.001). **Conclusions:** A positive teamwork climate is associated with higher levels of job satisfaction and improved perceptions of healthcare quality. These results suggest that hospital administrators should prioritize interventions that foster a supportive teamwork environment, as such efforts may enhance employees’ work attitudes and contribute to better healthcare quality.

## 1. Introduction

Since the 1990s, scholars have increasingly recognized the importance of interpersonal cooperation within healthcare organizations [1]. Hospital healthcare services depend on effective interdisciplinary collaboration among physicians, nurses, pharmacists, and administrative staff [2], and prior research has demonstrated that such collaboration is associated with improved healthcare quality [3,4,5,6]. Cooperation among healthcare professionals facilitates knowledge sharing, enhances problem-solving capabilities, reduces medical errors, and strengthens team functioning [7].

Addressing patients’ health concerns in complex healthcare systems requires seamless communication and coordination across departments [8]. Successful patient outcomes not only improve clinical performance but also enhance healthcare employees’ morale, organizational commitment, and sense of professional accomplishment [9,10]. Although existing studies have primarily focused on the direct effects of teamwork on care outcomes, organizational climate plays a critical role in shaping employees’ work attitudes and behaviors. Because healthcare delivery is largely mediated through interactions between healthcare staff and patients, employees’ attitudes and behaviors may directly influence the quality of care provided. Therefore, examining teamwork from a behavioral perspective is necessary to clarify whether teamwork climate affects employees’ work attitudes and, in turn, healthcare quality.

Previous research has largely attributed variations in healthcare quality to individual provider characteristics [11], such as clinical experience [12] and financial incentives [13]. While some studies have examined the relationships between teamwork, healthcare quality [14], and job satisfaction [15], the majority of this evidence has been derived from Western healthcare systems [16,17]. Empirical studies conducted in Germany [18,19] and the United States [20] have consistently reported positive associations between teamwork and job satisfaction. However, comparatively limited research has explored these relationships in Asian healthcare contexts.

An OECD survey on patient safety culture indicated that Western countries generally reported higher perceptions of teamwork, whereas Japan ranked 14th among Asian countries [21]. Given the structural, cultural, and organizational differences between Western and Asian healthcare systems, extending teamwork research to Asian settings is warranted. Although individual factors such as leadership have been shown to influence teamwork [22,23,24], organizational and psychological factors—particularly job satisfaction—remain insufficiently examined in relation to teamwork climate and healthcare quality [25,26,27].

Accordingly, this study explores the relationships among teamwork climate, job satisfaction, and healthcare quality in a Taiwanese hospital setting. By clarifying these relationships, this study aims to provide empirical evidence to inform hospital management strategies for fostering a positive teamwork climate and improving healthcare quality.

## 2. Literature Review

### 2.1. Relationship Between Teamwork Climate and Healthcare Quality

A team is commonly defined as a social system comprising three or more individuals embedded within an organizational context and working interdependently toward shared goals [28]. In healthcare settings, such interdependence is particularly critical, as patient care often requires coordinated decision-making and the integration of expertise from multiple professional groups. Accordingly, teamwork has been widely recognized as a foundational element in the delivery of safe and high-quality healthcare.

Sexton et al. conceptualized “teamwork climate” as employees’ shared perceptions of the quality of collaboration among healthcare personnel, reflecting the extent to which team members communicate openly, support one another, and coordinate their work effectively [29]. A positive teamwork climate facilitates timely information exchange, mutual trust, and shared accountability among physicians, nurses, and allied health professionals, thereby enabling more effective care coordination. Consistent with this perspective, the Institute of Medicine (IOM) emphasized team-based care as a collaborative approach in which multiple healthcare providers work collectively to optimize patient outcomes and reduce fragmentation in care delivery [30].

From an operational standpoint, effective teamwork enhances coordination across clinical roles, improves resource utilization, and reduces communication failures, which are a major source of medical errors [31]. By promoting shared understanding and coordinated action, a supportive teamwork climate contributes to safer care processes and more consistent clinical performance. Empirical evidence from OECD countries further supports this relationship, demonstrating that stronger teamwork is associated with higher levels of healthcare quality and lower patient mortality rates [32]. Taken together, theoretical perspectives and empirical findings suggest that teamwork climate plays a critical role in shaping healthcare quality.

The recent studies collectively also showed a consistent pattern: a more positive teamwork-related climate—whether reflected in direct measures of teamwork climate, teamwork perceptions, unit-level collaboration and communication, or a speaking-up climate—is generally associated with higher healthcare quality, higher patient safety grades, and better overall perceptions of patient safety [33,34,35,36].

Therefore, the following hypothesis is proposed:

**Hypothesis** **1 (H1).** *Teamwork climate is positively associated with healthcare quality*.

### 2.2. Relationship Between Teamwork Climate and Job Satisfaction

Job satisfaction is commonly defined as a positive emotional state resulting from an individual’s appraisal of their job or job-related experiences [37]. In healthcare organizations, job satisfaction is shaped by multiple job characteristics, including workload demands, organizational support, professional autonomy, and the quality of interpersonal relationships within the workplace [38]. Healthcare professionals routinely operate in high-pressure environments characterized by complex patient conditions, time constraints, and critical decision-making responsibilities. Under such conditions, working in isolation may intensify occupational stress and emotional exhaustion, thereby diminishing job satisfaction.

In contrast, a positive teamwork climate provides healthcare employees with social and professional support that can buffer the adverse effects of job-related stress. Effective teamwork facilitates knowledge sharing, collective problem-solving, and mutual assistance among team members, which can reduce uncertainty, minimize errors, and enhance confidence in clinical decision-making [39,40]. Moreover, a supportive teamwork climate fosters interpersonal trust, strengthens team cohesion, and promotes a sense of belonging within the organization. These psychosocial resources contribute to a more positive work environment and enhance employees’ perceptions of being valued and supported [41].

Overall, the literature published recently generally supports the same conclusion: a more positive teamwork climate or team-related climate is typically positively associated with higher job satisfaction. At the practice level, a more favorable team climate has been linked to higher job satisfaction, lower burnout, a greater likelihood of retention, and better patient experience ratings. These studies provide strong evidence for the association between team climate and job satisfaction [42,43,44,45].

Consequently, when healthcare employees perceive a strong teamwork climate, they are more likely to experience higher levels of job satisfaction due to reduced work stress, improved collaboration, and enhanced interpersonal relationships. Based on this theoretical and empirical evidence, the following hypothesis is proposed:

**Hypothesis** **2 (H2).** *Teamwork climate is positively associated with employees’ job satisfaction*.

## 3. Methods

### 3.1. Study Design

This study adopted a cross-sectional survey design to examine the correlation of hospital employees’ perceptions of teamwork climate, job satisfaction, and healthcare quality.

### 3.2. Sample and Setting

In Taiwan, hospitals are classified as medical centers, regional hospitals, or district hospitals based on resource availability and service capacity [46]. Regional hospitals, which are subject to specific accreditation requirements, typically provide a broad range of medical services and possess relatively comprehensive clinical resources. Participants were recruited from a regional teaching hospital located in northern Taiwan. All 528 healthcare professionals of the hospital, including physicians (N = 104), nurses (N = 309), and medical technicians and others (N = 115), were invited to participate in the study. The questionnaires were distributed and collected directly by the research staff. Of the 528 questionnaires distributed, 465 were returned, and 462 were deemed valid for analysis, resulting in a valid response rate of 87.5%. Missing data were addressed using the hot deck imputation method [47]. Hot deck imputation is a commonly used method in survey data processing in the social sciences. Because attitudes are expected to exhibit some consistency, this approach helps preserve the distributional characteristics of the data.

### 3.3. Measurement Instruments

#### 3.3.1. Teamwork Climate and Job Satisfaction

Teamwork climate and job satisfaction were measured using selected subscales of the Safety Attitudes Questionnaire (SAQ), originally developed by Gabrani et al. [48] and subsequently revised by Sexton et al. [49]. The SAQ has been widely validated across diverse healthcare settings, including in the United States, the United Kingdom, and New Zealand [50], and has been translated into Chinese and psychometrically tested in Taiwan [51].

In this study, teamwork climate was assessed using six items, and job satisfaction was measured using five items from the validated SAQ instrument. Responses were recorded on a 5-point Likert scale ranging from 1 (strongly disagree) to 5 (strongly agree), with higher scores indicating more positive perceptions.

#### 3.3.2. Healthcare Quality

Healthcare quality was assessed based on Donabedian’s framework, which conceptualizes quality of care across three dimensions: structure (e.g., resources, tools, and staff training), process (e.g., care delivery practices and patient–provider interactions), and outcomes (e.g., perceived improvements in patient health status) [52]. A 16-item scale was developed to measure these dimensions. The instrument was evaluated and refined through a pilot study prior to the main data collection. Responses were measured using a 5-point Likert scale ranging from 1 (strongly disagree) to 5 (strongly agree), with higher scores reflecting more favorable perceptions of healthcare quality.

### 3.4. Pilot Study and Reliability

A pilot study was conducted with 50 healthcare professionals to assess the reliability and clarity of the questionnaire. During the pre-test, one negatively worded item in the teamwork climate subscale exhibited a low correlation with the total scale score (Spearman’s ρ = 0.238) and was therefore removed. And two items of Healthcare Quality were also removed because of low factor loadings. Following this revision, the final questionnaire demonstrated strong internal consistency. Cronbach’s α coefficients were 0.852 for teamwork climate, 0.923 for job satisfaction, and 0.945 for healthcare quality, all exceeding the recommended threshold of 0.70 and indicating high reliability [50].

### 3.5. Data Analysis

Data analyses were performed using SPSS for Windows, Version 22.0 (IBM Corp., Armonk, NY, USA). Descriptive statistics were calculated to summarize sample characteristics, including means and standard deviations. Correlation analyses were conducted to examine the relationships among teamwork climate, job satisfaction, and healthcare quality. One-way analysis of variance (ANOVA) was used to assess differences in perceptions of teamwork climate, job satisfaction, and healthcare quality across demographic groups. Exploratory factor analysis was performed to evaluate the construct validity of the measurement instruments.

## 4. Results

### 4.1. Participants’ Characteristics

A total of 462 hospital employees completed the survey, yielding a valid response rate of 87.5%. The majority of respondents were female (81.8%) and aged between 31 and 40 years (35.5%). Most participants did not hold supervisory or managerial positions (87.7%). In terms of professional role, over half of the respondents were nurses (50.9%), followed by physicians and medical technicians. With respect to educational attainment, most participants held a college or university degree (80.8%). Regarding work experience, 65.2% reported more than five years of service in their current organization. The majority of respondents were employed on a full-time basis (97.0%). Detailed demographic characteristics of the study sample are presented in Table 1.

### 4.2. Validity and Reliability of the Questionnaire

Table 2 summarizes the descriptive statistics, exploratory factor analysis (EFA) results, and internal consistency of the study variables. The Cronbach’s α coefficients for teamwork climate, job satisfaction, and healthcare quality were 0.852, 0.935, and 0.965, respectively, indicating strong internal reliability.

Exploratory factor analysis was conducted using principal component analysis with varimax rotation, assuming three uncorrelated factors [53]. Items with factor loadings below 0.60 or cross-loadings greater than 0.40 were excluded. Following EFA, both teamwork climate and job satisfaction each retained a single-factor structure comprising five items. Healthcare quality was represented by two dimensions—quality of care structure and process (QC) and quality of care outcomes (QO)—with a total of 14 retained items.

Consistent with the recommendations of Hair et al. [53], the summated scale approach was applied. No meaningful differences were observed between orthogonal and oblique rotation solutions. All retained items exhibited factor loadings greater than 0.70, and inter-item correlations exceeded 0.30, satisfying criteria for convergent validity. Discriminant validity was evaluated by comparing the square roots of the average variance extracted (AVE) with the inter-construct correlations. As presented in Table 2, the square roots of the AVE values (shown on the diagonal in bold) exceeded the corresponding correlation coefficients for all constructs. Although a marginal overlap was observed between the QC and QO dimensions (*p* < 0.10) [54,55], the overall pattern supported adequate discriminant validity. Although QC and QO were moderately correlated, the correlation coefficient did not exceed the commonly accepted threshold indicating serious multicollinearity. Regarding discriminant validity, because this study adopted EFA, the two dimensions were retained based on their empirical factor structure and item loadings. Although a marginal overlap was observed, the retained items loaded primarily on their respective factors, supporting the conceptual distinction between QC and QO.

### 4.3. Relationship Between Teamwork Climate, Job Satisfaction, and Healthcare Quality

Among the study variables, healthcare quality exhibited the highest mean score (M = 4.04, SD = 0.59), followed by teamwork climate (M = 3.98, SD = 0.75) and job satisfaction (M = 3.84, SD = 0.77) (Table 3). Correlation analyses revealed significant positive associations among the study variables. Teamwork climate was positively associated with job satisfaction (*r* = 0.695, *p* < 0.001) and healthcare quality (*r* = 0.433, *p* < 0.001). Moreover, job satisfaction was positively associated with healthcare quality (*r* = 0.497, *p* < 0.001). These findings provide empirical support for Hypotheses 1 and 2, indicating that a supportive teamwork climate contributes both to higher employee job satisfaction and to improved perceptions of healthcare quality.

Results from one-way analysis of variance (ANOVA) indicated that gender was significantly associated with perceptions of teamwork climate (*p* = 0.035). Age demonstrated a significant association with job satisfaction (*p* < 0.001) and healthcare quality (*p* = 0.004) (Table 1).

Leadership status (leader vs. non-leader) was not significantly associated with teamwork climate, job satisfaction, or healthcare quality (*p* > 0.05). Education showed significant differences in job satisfaction (*p* < 0.01) and healthcare quality (*p* < 0.05), but not in teamwork climate (*p* > 0.05). In addition, neither length of service at the hospital nor employment status (full-time vs. part-time) was significantly associated with teamwork climate, job satisfaction, or healthcare quality (*p* > 0.05) (Table 1).

## 5. Discussion

### 5.1. Influence of Demographics on Teamwork Climate, Job Satisfaction, and Healthcare Quality

The present study revealed that certain demographic factors were significantly associated with perceptions of teamwork climate, job satisfaction, and healthcare quality. Gender was significantly associated with teamwork climate, with female employees reporting higher perceptions than male employees. Age was significantly associated with job satisfaction and healthcare quality, with older employees reporting higher satisfaction and perceived care quality, likely reflecting accumulated professional experience and greater adaptation to organizational practices.

Education also influenced key outcomes. Employees with higher educational attainment, such as Master’s degrees, often occupy administrative or managerial roles requiring enhanced communication, coordination, and leadership skills, which may contribute to higher perceptions of teamwork climate. Additionally, higher education levels are typically associated with greater financial compensation and career advancement opportunities, which may further enhance job satisfaction.

Differences across professional roles were also observed. Nurses reported lower job satisfaction than physicians, but higher than technicians and other staff. While previous studies in Taiwan have indicated lower job satisfaction among nurses compared with other healthcare professionals [56,57], the present findings suggest a more nuanced pattern that warrants further investigation in future research.

### 5.2. Impact of Teamwork Climate on Job Satisfaction and Healthcare Quality

Consistent with Hypothesis 1, the present study demonstrates that teamwork climate is positively associated with healthcare quality. Employees who perceive a supportive and collaborative team environment report higher-quality care, suggesting that effective coordination, knowledge sharing, and communication among healthcare personnel directly contribute to improved patient outcomes. These findings align with prior research indicating that cohesive team environments enhance clinical performance and reduce errors [39,56,58].

In addition, consistent with Hypothesis 2, teamwork climate was found to positively influence employees’ job satisfaction. A positive teamwork climate provides healthcare staff with social and professional support, fosters trust and cohesion among team members, and facilitates shared problem-solving. These mechanisms contribute to a more favorable work environment and enhance employees’ perceptions of their job roles, thereby increasing job satisfaction [39,58]. Taken together, these results indicate that teamwork climate plays a dual role in hospital settings: it directly improves healthcare quality (H1) and simultaneously enhances employee job satisfaction (H2), which may further reinforce care quality through increased engagement and motivation. From a managerial perspective, these findings underscore the importance of interventions aimed at fostering a positive teamwork climate. Strategies may include promoting job autonomy, enhancing task significance, providing professional development opportunities, implementing structured communication protocols, and supporting collaborative leadership practices [59].

Furthermore, the results highlight the importance of considering team composition and interprofessional dynamics when designing such interventions. Effective communication, mutual support, and coordination among diverse healthcare professionals are critical for both employee satisfaction and patient care quality [39,60]. Future research should examine how factors such as leadership style, team diversity, and communication models may strengthen the relationship between teamwork climate, job satisfaction, and healthcare quality, thereby providing more nuanced insights into the mechanisms underpinning H1 and H2.

### 5.3. Limitations

The present study has some limitations. Because causal inference is not easy to establish, we revised the analysis to focus on correlations instead. The participants in this study had higher scores for healthcare quality than for other dimensions. However, we examined only one hospital; hence, our findings may not be well generalizable to other hospitals. But because the internal structures of hospitals in Taiwan are generally similar, and hospital operations are influenced by the National Health Insurance system, the content of services may vary by size and accreditation level, but they remain highly comparable overall. Therefore, although this study used data from only a single hospital, the findings still have reference value. In measuring healthcare quality, an analysis based on actual clinical care performance indicators would provide a more direct assessment. However, because healthcare quality indicators involve a wide range of dimensions, this study instead focused on employees’ perceptions of in-hospital quality management practices and culture. In future research, we may incorporate actual clinical care indicators into the analysis. Our investigation focused on hospital employees but did not compare interprofessional team operations to measure the effect of team diversity. Future studies should examine medical teams in various clinical departments and hospitals of different sizes to examine the effects of team characteristics or hospital sizes on team operations. In addition, teamwork is a dynamic behavior, and our cross-sectional study failed to observe the dynamic processes of teams. Hence, longitudinal studies should examine how team operations affect healthcare quality.

## 6. Conclusions

The findings of this study suggest that teamwork climate is highly important for hospital employees. When employees perceive stronger management support, better team communication, greater supervisory support for patient safety, and unit characteristics such as mutual cooperation, they are also more likely to give the hospital a favorable evaluation of patient safety. In a positive work climate, employees are also more likely to feel supported by and helpful toward one another, which in turn contributes to higher job satisfaction. This is especially important in hospitals, where services directly affect patients’ lives, staff must constantly work against time, and a high level of vigilance and tension is always required.

The primary objective of healthcare services is to provide high-quality care and satisfaction to patients. The present study showed that, among healthcare staff, teamwork climate and employees’ job satisfaction influence healthcare quality. Therefore, hospital managers can improve the quality of care provided to patients by increasing the job satisfaction of healthcare staff.

## Figures and Tables

**Table 1:** Participant Characteristics and One-Way ANOVA Analysis. N = 462.

Dependent Variables	Independent Variable	N (%)	Mean	S.D.	F Value/ T Value	*p* Value
Teamwork climate	**Gender**				4.449	0.035 *
	Female	378 (81.8)	4.12	0.89		
	Male	84 (18.2)	3.95	0.71		
	**Age**				1.487	0.217
	≤30	99 (21.4)	3.84	0.86		
	31–40	164 (35.5)	4.03	0.68		
	41–50	112 (24.2)	3.99	0.68		
	≥51	87 (18.8)	4.02	0.81		
	**Leader/Non-leader**				0.045	0.833
	Leader	57 (12.3)	4.14	0.82		
	Non-leader	405 (87.7)	3.95	0.74		
	**Department**				1.016	0.363
	Physician	82 (17.7)	4.04	0.83		
	Technician and others	145 (31.4)	3.93	0.77		
	Nurse	235 (50.9)	4.02	0.67		
	**Education**				4.39	0.013 *
	Higher school	55 (11.9)	3.94	0.72		
	College/University	373 (80.7)	3.95	0.77		
	Master	34 (7.4)	4.34	0.54		
	**Length of service at this hospital**				1.081	0.340
	<1 year	27 (5.8)	3.81	0.71		
	1 year–4 year	134 (29.0)	3.94	0.79		
	≥5 year	301 (65.2)	4.01	0.74		
	**Full-time/Part-time**				0.001	0.978
	Full-time	448 (97.0)	3.99			
	Part-time	14 (3.0)	3.59			
Job Satisfaction	**Gender**				3.363	0.067
	Female	378 (81.8)	4.05	0.89		
	Male	84 (18.2)	4.79	0.74		
	**Age**				7.031	<0.001 **
	21–30	99 (21.4)	3.58	0.84		
	31–40	164 (35.5)	3.82	0.72		
	41–50	112 (24.2)	3.91	0.71		
	≥51	87 (18.8)	4.08	0.79		
	**Leader/Non-leader**				0.020	0.887
	Leader	57 (12.3)	4.13	0.79		
	Non-leader	405 (87.7)	3.80	0.76		
	**Department**				6.871	<0.001 **
	Physician	82 (17.7)	4.03	0.83		
	Technician and others	145 (31.4)	3.71	0.78		
	Nurse	235 (50.9)	3.94	0.69		
	**Education**				5.321	0.05 *
	Higher school	55 (11.9)	3.96	0.66		
	College/University	373 (80.7)	3.79	0.79		
	Master	34 (7.4)	4.20	0.64		
	**Length of service at this hospital**				0.262	0.770
	<1 year	27 (5.8)	3.78	0.63		
	1 year–4 year	134 (29.0)	3.81	0.80		
	≥5 year	301 (65.2)	3.86	0.78		
	**Full-time/Part-time**				0.216	0.642
	Full-time	448 (97.0)	3.85			
	Part-time	14 (3.0)	3.47			
Healthcare Quality	**Gender**				2.788	0.096
	Female	378 (81.8)	4.26	0.59		
	Male	84 (18.2)	3.99	0.58		
	**Age**				4.525	0.004 **
	21–30	99 (21.4)	3.94	0.67		
	31–40	164 (35.5)	3.95	0.55		
	41–50	112 (24.2)	4.15	0.55		
	≥51	87 (18.8)	4.15	0.57		
	**Leader/Non-leader**				0.012	0.914
	Leader	57 (12.3)	4.27	0.54		
	Non-leader	405 (87.7)	4.00	0.59		
	**Department**				3.613	0.028 *
	Physician	82 (17.7)	4.12	0.57		
	Technician and others	145 (31.4)	3.96	0.61		
	Nurse	235 (50.9)	4.10	0.56		
	**Education**				4.141	0.017 *
	Higher school	55 (11.9)	3.95	0.58		
	College/University	373 (80.7)	4.02	0.58		
	Master	34 (7.4)	4.30	0.62		
	**Length of service at this hospital**				0.348	0.706
	<1 year	27 (5.8)	4.03	0.59		
	1 year–4 year	134 (29.0)	4.00	0.65		
	≥5 year	301 (65.2)	4.05	0.56		
	**Full-time/Part-time**				0.005	0.942
	Full-time	448 (97.0)	4.04			
	Part-time	14 (3.0)	3.81			

Note: * *p <* 0.05, ** *p <* 0.01.

**Table 2:** Results of factor analysis and reliability.

Scales Constructs/Content of Items	Mean	SD	Factor Loading	% of Variance	Cronbach’s α	AVE
**Teamwork climate (TC)**	3.977	0.751		**14.819%**	**0.852**	**0.525**
It is easy for employees to ask questions when they do not understand something.	3.781	0.982	0.723			
I receive support from another colleague to care for patients.	3.848	1.022	0.821			
Healthcare staff input is well-received in this hospital.	3.985	0.877	0.868			
Disagreements in this hospital are resolved appropriately (i.e., not according to who is right but according to what is best for the patient).	4.277	0.870	0.823			
The employees work together as a well-coordinated team.	3.991	0.977	0.743			
**Job Satisfaction (JS)**	3.838	0.751		**16.201%**	**0.935**	**0.611**
I like my job.	3.751	0.874	0.697			
This hospital is a good workplace.	4.006	0.837	0.771			
Our team provides a good work environment.	3.870	0.877	0.853			
I am proud to work at this hospital.	3.874	0.870	0.869			
This hospital has high morale.	3.688	0.880	0.782			
**Healthcare Quality (HQ)**	4.036	0.590				
***The construct of healthcare (QC)***	4.022	0.614		***19.589%***	***0.931***	***0.523***
Our hospital has a clear vision regarding healthcare quality.	3.859	0.809	0.774			
Our hospital’s managers facilitate or contribute to the quality of healthcare services.	3.931	0.745	0.814			
Our hospital has a reward system to encourage quality improvement activities.	4.000	0.782	0.769			
Our hospital provides the required resources to undertake quality improvement activities.	3.965	0.747	0.766			
Our hospital has a system for reporting incidental events.	4.253	0.651	0.696			
Our hospital provides quality-of-care training and education to employees.	4.069	0.694	0.712			
The healthcare staff are guaranteed to maintain the quality of care for patients.	4.078	0.670	0.711			
***The outcome of healthcare (QO)***	4.049	0.632		***22.875%***	***0.947***	***0.596***
Our hospital’s employees communicate well with others.	3.970	0.770	0.756			
The healthcare staff cooperates well at our hospital.	4.030	0.736	0.796			
The healthcare staff communicates with patients well.	4.106	0.700	0.779			
The healthcare staff have professional skills to care for patients.	4.141	0.700	0.783			
Patients are satisfied with the quality of healthcare at our hospital	4.017	0.712	0.802			
The healthcare staff commits to patients’ health.	4.080	0.700	0.805			
We provide healthcare to patients through teamwork.	4.000	0.757	0.759			

Note: The KMO value of all items factor analysis was 0.947. Cumulative variance % was 73.384. AVE: Average Variance Extracted.

**Table 3:** The relationship between teamwork, job satisfaction, and healthcare quality.

Construct	Descriptive Statistic			Correlation Coefficients (r)		
	Means	SD	n	TC	JS	HQ
Teamwork Climate (TC)	3.977	0.751	462	**1**	0.695 **	0.433 **
Job Satisfaction (JS)	3.838	0.773	462	0.695 **	**1**	0.497 **
Healthcare Quality (HQ)	4.039	0.586	462	0.433 **	0.497 **	**1**

** *p*-value < 0.001.

## Data Availability

The raw data supporting the conclusions of this article will be made available by the authors upon request.
